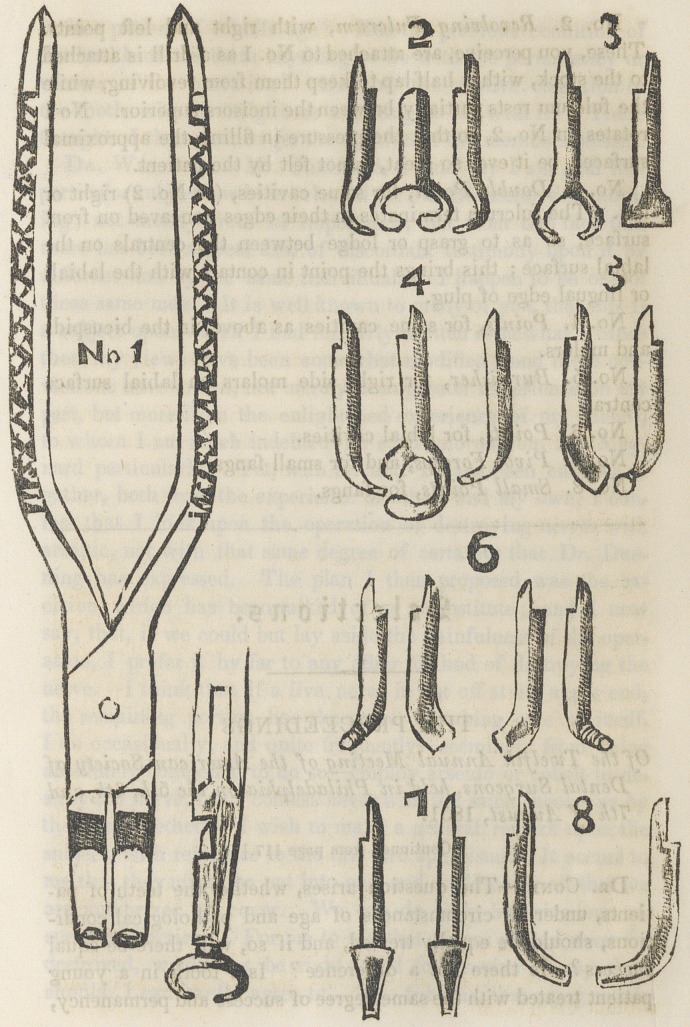# Dr. Baxter’s Condensing Forceps

**Published:** 1852-04

**Authors:** 


### DR. BAXTER'S CONDENSING FORCEPS.

We give the drawing and description of these ingeniously contrived and admirably adapted instruments. Dr. Baxter exhibited these at the last meeting of our society; and by referring to the proceedings of the association it will be perceived a commendatory resolution was passed in relation to them.

The wood cut presents rather broader page than the Register, but arrived too late to be altered. ED.

WARSAW, KY., MARCH 25, 1852.

DR. TAYLOR: I send you cut of my forceps, with the movable points for condensing the plugs after they are introduced with a common plugger. In using the instrument care should be taken to shield the opposite surface of the tooth with cotton or a napkin. I find the instrument to be of great utility, in fact I could hardly do without it. The first one I used I made out of a pair of round-nosed plyers, three years ago, and still it can be improved on. I have omitted the double forceps for want of room on the block, but I will engrave it some other time. This double instrument has three beaks, two grasp the molar, whilst the other point condenses the plug in the centre cavity. This makes it a complete instrument, and adapted to any cavity.

DESCRIPTION OF THE CUTS, PAGE 171.

No. 1. *The Handle,* showing the side and edge, with, and without the points.

No 1
2
3
4
5
6
7
8

No. 2. *Revolving Fulcrum*, with right and left points. These, you perceive, are attached to No. 1 as a drill is attached to the stock, with a half lap to keep them from revolving, while the fulcrum rests partially between the incisors superior. No 1 rotates on No. 2, so that the pressure in filling the approximal surfaces, be it ever so great, is not felt by the patient.

No. 3. *Double Point*, for same cavities, (as No. 2) right or left. The fulcrum terminates in their edges concaved on front surface, so as to grasp or lodge between the centrals on the labial surface ; this brings the point in contact with the labial, or lingual edge of plug.

No. 4. *Points*, for same cavities as above, in the bicuspids and molars.

No. 5. *Burnisher*, for right side molars on labial surface central.

No. 6. *Points*, for labial cavities.

No. 7. *Pivot Forceps*, and for small fangs.

No. 8. *Small Points*, for fangs.

## Figures and Tables

**Figure f1:**